# Incidence of Poor Sleep Quality and Its Predictors Among Adults With Upper Gastrointestinal Symptoms Visiting a Tertiary Care Hospital in Central India

**DOI:** 10.7759/cureus.92600

**Published:** 2025-09-18

**Authors:** Kritika Singhal, Vindhya Solanki, Rashida Ali, Roshan F Sutar, Abhijit Rozatkar, Pankaj Prasad, Surya Bali, Deepti Dabar, Mohit Kumar, Anindo Majumdar

**Affiliations:** 1 Community Medicine, Ram Krishna Medical College Hospital and Research Centre, Bhopal, IND; 2 Community and Family Medicine, All India Institute of Medical Sciences, Bhopal, Bhopal, IND; 3 Psychiatry, All India Institute of Medical Sciences, Bhopal, Bhopal, IND

**Keywords:** anxiety, depression, gastroesophageal reflux, sleep problems, sleep quality and quantity

## Abstract

Background

Sleep disturbances are frequently associated with upper gastrointestinal (GI) symptoms, including gastroesophageal reflux disease (GERD). However, data from Indian settings remain limited. This study aimed to assess the proportion and predictors of poor sleep quality among adults with upper GI symptoms.

Methodology

A hospital-based, cross-sectional study was conducted at a tertiary care center in Bhopal, India, from November 2019 onwards. Adults presenting with upper GI symptoms were assessed using the GERD-Q, Patient Health Questionnaire-9 (PHQ-9), Generalized Anxiety Disorder-7 (GAD-7), and Pittsburgh Sleep Quality Index (PSQI). Univariable and multivariable logistic regression analyses were used to identify predictors of poor sleep quality (PSQI score >5).

Results

Among 500 participants (mean age = 38.1 ± 14.7 years; 62.6% male), 69.9% (306/500) had poor sleep quality. Female gender (adjusted odds ratio (aOR) = 2.07, p = 0.017), tobacco use (aOR = 2.47, p = 0.003), being underweight (body mass index <18.5 kg/m²; aOR = 2.43, p = 0.043), and moderate depression (PHQ-9 score of 10-14; aOR = 2.64, p = 0.033) were significant predictors (N = 306/500). While GERD symptoms were common, GERD diagnosis (GERD-Q score ≥8) was not an independent predictor after adjustment.

Conclusions

Poor sleep quality is prevalent among patients with upper GI symptoms and is influenced by gender, lifestyle, nutritional status, and psychological well-being rather than GERD alone. Integrated care approaches addressing these factors are essential for improving sleep health in this population.

## Introduction

Sleep disturbances are linked to upper gastrointestinal (GI) symptoms and nocturnal gastroesophageal reflux (GER), which has, in turn, also been associated with poor sleep quality [1]. Studies have shown that upper GI symptoms significantly impact daily quality of life. Common complaints reported in outpatient clinics include nighttime GER symptoms, insomnia, and poor-quality sleep, all of which interfere with rest and well-being [2].

Cremonini et al. conducted a study aiming to determine whether there is an association between self-reported sleep disturbances and frequent upper and lower GI symptoms in the community. The participants reported that there was significant sleep disturbance with the presence of upper GI symptoms such as bloating, nausea, acid regurgitation, and heartburn, in which the participants reported waking up tired at least eight times a month. They also reported significant disturbance in falling and staying asleep [3].

Shaker et al. conducted a survey of 1,000 adults with heartburn at least twice weekly and found that 79% of respondents had nighttime symptoms. Of these, 75% had symptoms that affected sleep, 63% thought that heartburn affected their ability to sleep well, and 40% reported that these interfering symptoms diminished their ability to function well the next day. These data indicate a clear association between reflux symptoms and poor sleep quality [4].

Accompanying these changes are sleep-related decreases in the activity of skeletal muscles, including the cricopharyngeal and diaphragm muscles. These two muscles contribute to the pressure generated within the upper and lower esophageal sphincters (UES and LES), respectively. An important role of these sphincters is to prevent regurgitation of gastric contents into the esophagus (GER) in the case of the LES, and regurgitation and potential aspiration of esophageal contents into the larynx and pharynx (pharyngoesophageal reflux) in the case of the UES. Multiple studies have shown that sleep has major effects on gastroesophageal function, including decreased swallowing, saliva production, and esophageal motor activity [5-7].

Bajaj et al. reported a progressive decline in UES pressure with deeper sleep stages, reaching its lowest during slow wave sleep (SWS), while Kahrilas et al. found no significant differences across sleep stages [8,9]. Regarding the LES, Avots-Avotins et al. observed no significant day-night differences in pressure, and Dent et al. noted substantial variability during sleep, though neither examined changes across specific sleep stages [10,11]. Transient lower esophageal sphincter relaxation, a vasovagal response to gastric distention, is the main reflux mechanism in both health and disease, and becomes more problematic when coupled with factors such as hypotensive LES or hiatal hernia [12]. Eastwood et al. demonstrated that UES pressure and barrier function decrease during sleep, especially during SWS and expiration, making it more prone to pharyngoesophageal reflux [13].

The evidence clearly suggests that upper gastroesophageal symptoms have a physiological basis of worsening during sleep and impact the quality of sleep. There is a dearth of studies from India reporting the association between sleep quality and upper GI symptoms. Hence, we conducted this study to determine (a) the incidence of poor sleep quality among adult patients suffering from symptoms of upper GI disorders; (b) sociodemographic predictors of poor sleep quality among them; (c) lifestyle predictors (diet, exercise, tobacco and alcohol consumption) of poor sleep quality among them; and (d) psychological predictors (depression and anxiety) of poor sleep quality among them.

## Materials and methods

This hospital-based, cross-sectional study was conducted in the general outpatient clinic run by the Department of Community and Family Medicine, All India Institute of Medical Sciences, Bhopal. This clinic functions in a tertiary care hospital attached to a medical college in Bhopal, the capital city district of Madhya Pradesh state of India. The hospital is located in an urban area of the district and caters to people living in both urban and rural areas of the state and nearby regions of central India. Data collection was started in November 2019 and completed in February 2023.

The study participants were consecutively recruited and included all patients visiting the clinic with symptoms of upper GI disorders during the study period. All adult patients (above the age of 18 years) reporting symptoms of upper GI disorders and willing to participate in the study were included. Patients with established (diagnosed/on treatment) hypertension, diabetes, chronic respiratory diseases, cardiovascular disease, moderate-to-severe anemia, thyroid disorders, and any established neurological condition were excluded from the study.

Operational definition of symptoms of upper GI disorders was followed as per the International Foundation for Gastrointestinal Disorders. These were heartburn, difficulty swallowing, stomach pain, nausea, vomiting, problems in the passage of food, or any combination of these symptoms.

**Figure 1:**
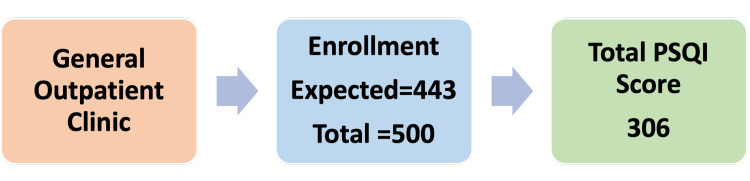
Patient flow diagram. The PSQI score was available only for 306 participants, as the sleep latency score item was completed only by 306 participants. Overall, 443 was the expected enrolment based on the sample size calculation. PSQI = Pittsburgh Sleep Quality Index

We enrolled a total of 500 participants as they presented to the outpatient department during the data collection to account for any statistical corrections required, such as missing data, and as logistical availability allowed. According to previous literature, the prevalence of poor sleep quality in such populations ranges from 50% to 70% [3,4]. Assuming an expected prevalence of 60%, with a 5% absolute precision and a 95% confidence level, the minimum required sample size was calculated using the following formula for single proportion estimates for cross-sectional studies: n = z^2 ^× p(1-p)/d^2^; on substituting the values: z = 1.96, p = 0.60 and d = 0.05, the total calculated sample size was 369 participants. Assuming a 20% non-response rate, the calculated minimum sample size was 443 (Figure 1).

In addition, a priori power analysis was conducted to ensure adequate power for the secondary objective of identifying predictors of poor sleep quality using multivariable logistic regression. Using G*Power 3.1 software, and assuming a moderate effect size (odds ratio (OR) = 1.7-2.0), alpha level of 0.05, power of 80%, and approximately 10 predictors, the minimum required sample size for regression was estimated to be between 300 and 350 participants. As 306 participants had complete data on the Pittsburgh Sleep Quality Index (PSQI) and were included in the final regression model, the study had adequate power to detect significant associations between predictors and sleep quality.

Pretested semi-structured interview schedules were developed, and previously validated available tools were used to collect information regarding the sociodemographic profile of the participants, clinical history and details, presence of symptoms of gastroesophageal reflux disease (GERD), and sleep quality. The tools were translated, whenever applicable, in the local language (Hindi), if they were not already available in Hindi. Height and weight were measured using the routinely used clinic’s stadiometer and electronic weighing scale using standard procedures. Body mass index (BMI) was calculated. Data were collected by two co-authors with the help of treating physicians posted in the clinic. Exit interviews of the patients after their consultation with the physician for the condition for which they had visited were conducted. Before the commencement of the study, all physicians were sensitized and briefed regarding the study, including a session on recognizing the symptoms of upper GI disorders.

The Gastroesophageal Reflux Disease Questionnaire (GERD-Q) tool was used to diagnose GERD. GERD-Q is a six-item, easy-to-use questionnaire that was developed primarily as a diagnostic tool for GERD in primary care patients consulting for upper GI complaints [14]. This questionnaire was developed by Jones et al. in 2009 for the diagnosis and management of GERD patients. It was created from three different validated questionnaires evaluated in the DIAMOND study. This questionnaire consists of six items, which include four positive predictors of GERD, including heartburn, regurgitation, sleep disturbance, and use of over-the-counter medications. The remaining two items are negative predictors, which include epigastric pain and nausea. Patients were asked to reflect on symptoms over the preceding week. Scores ranging from 0 to 3 were applied for the positive predictors and from 3 to 0 (reversed order, where 3 = none) for negative predictors. The score was calculated as the sum of these scores, giving a total score ranging from 0 to 18, and the optimal balance between sensitivity and specificity was achieved when using a cut-off value ≥8, which was also used to define the presence of GERD in this study. This tool is freely available for clinical and research use as stated by its developers, and no formal permission is required for non-commercial academic use [15]. This questionnaire was applied in a study conducted in southern India in 2011 to assess the prevalence and risk factors of GERD [16]. In our study, interviewers were trained to ask patients these questions in either English or a translated Hindi version based on the preferred language choice of the patient. This study is available for free on the internet. This tool is available online for use in research and publication without the need for permission, as confirmed by relevant authorities at AstraZeneca.

The Patient Health Questionnaire-9 (PHQ-9), a self-administered questionnaire, was used to assess depression in the present study. This tool consists of nine items, which are symptoms of depression assessed in terms of duration for which they are experienced, i.e., not at all (0), several days (1), more than half the days (2), and nearly every day (3). The total score ranges from 0 to 27, where 1-4 is minimal, 5-9 is mild, 10-14 is moderate, 15-19 is moderately severe, and 20-27 is severe depression [17]. This questionnaire was validated in multiple studies conducted in India using focus group discussions, qualitative interviews, and expert reviews. The difficult phrases in the tool were changed, and the length of two items was shortened to aid comprehension. Patients whose PHQ-9 score exceeded the cut-off scores of 10 were referred for diagnostic evaluation and further appropriate management to the outpatient department of Psychiatry [18,19].

The Generalized Anxiety Disorder-7 (GAD-7) tool, a self-administered questionnaire, was used to assess anxiety in the present study. This tool consists of seven items, which are symptoms of anxiety assessed in terms of duration for which they are experienced, i.e., not at all (0), several days (1), more than half the days (2), and nearly every day (3). The total score ranges from 0 to 21, where 0-4 is minimal anxiety, 5-9 is mild anxiety, 10-14 is moderate anxiety, and 15-21 is severe anxiety [20]. This tool has been translated into Hindi and has been validated in Indian settings [21]. This tool is available for free on the internet.

PHQ-9 and GAD-7 are in the public domain. They are available free of charge for clinical and research purposes without the need for special permissions, as confirmed by Spitzer et al., authors and Pfizer Inc., which have made the tools publicly accessible [17,20].

The PSQI was used to study the sleep quality. It is also a self-administered questionnaire with seven domains (sleep latency, sleep duration, subjective sleep quality, sleep efficiency, sleep disturbance, daytime dysfunction, and sleep medication use) containing 19 items in total. Each domain has a different number of questions, which are rated individually. The scores from the seven domains are added to give a global score ranging from 0 to 21 (no to severe difficulties). Further, a global PSQI score of more than 5 indicates a poor quality of sleep [22]. This tool has been translated into Hindi and has been validated in Indian settings [23]. This tool is available for non-commercial research as per the information on the website of the Department of Psychiatry, Centre for Sleep and Circadian Science, University of Pittsburgh. Permissions are required only for commercial research purposes. Additionally, the link to the English version of PSQI in an academic publication is free of cost.

Ethical approval for the study was received from the Institutional Human Ethics Committee (IHEC) of All India Institute of Medical Sciences (AIIMS), Bhopal (reference number: IHEC-LOP/2019/IM0213). Written Informed consent was taken from each participant before enrolling in the study.

Data collection was done with the help of Kobo Toolbox. The data were analyzed using SPSS software (IBM Corp., Armonk, NY, USA). Categorical data were presented as frequencies and percentages. Chi-square test and Fisher’s exact test were used to determine the association between categorical variables. Univariable and multivariable logistic regression analyses were used to determine the predictors of sleep quality. The variables that had a p-value <0.25 in univariable analysis were included in the multivariable model. Unadjusted and adjusted ORs were reported, respectively. All tests were conducted with a 95% confidence interval (CI) and a significant p-value of 0.05. Any missing data was removed from the analysis.

## Results

A total of 500 participants completed the questionnaire. Table 1 depicts the sociodemographic, behavioral, anthropometrical, and clinical parameters of the participants. The mean age of the participants was 38.1 ± 14.7 years. There were 313 (62.6%) males and 187 (37.4%) females. More than half of the participants were residents of urban areas (301, 60.20%) and were married (341, 68.20%). Further, about two-thirds of participants (337, 67.40%) belonged to the upper socioeconomic status according to the revised BG Prasad scale. More than half of the participants belonged to nuclear families (282, 56.40%). As for substance use, 224 (44.80%) participants consumed tobacco, and 137 (27.40%) consumed alcohol. Almost one-third of the participants (151, 30.20%) agreed to getting sufficient exercise, and 297 (59.40%) participants were vegetarians. A total of 101 (20.20%) participants were underweight, 194 (38.80%) were normal and overweight, respectively, and 11 (2.20%) were obese according to the WHO Asian criteria for BMI [24].

**Table 1:** Sociodemographic, behavioral, and clinical characteristics of the study participants. ^#^: Mean ± standard deviation.

Sociodemographic, behavioral, and clinical characteristics	N (%) (n = 500)
Age^#^ (years)	38.09 ± 14.66
Gender	
Male	313 (62.60)
Female	187 (37.40)
Residence	
Urban	301 (60.20)
Rural	199 (39.80)
Occupation	
Arithmetic skills job	46 (9.20)
Skilled worker	129 (25.80)
Semi-skilled worker	10 (2.00)
Professional	48 (9.60)
Semi-professional	43 (8.60)
Unskilled worker	4 (0.80)
Retired	4 (0.80)
Housewife	113 (22.60)
Unemployed	103 (20.60)
Education	
Postgraduate	34 (6.80)
Graduate	158 (31.60)
Higher secondary	133 (26.60)
High school	71 (14.20)
Middle school	25 (5.00)
Literate	10 (2.00)
Illiterate	69 (13.80)
Socioeconomic status	
I (upper)	337 (67.40)
II (upper middle)	110 (22.00)
III (middle)	29 (5.80)
IV (lower middle)	14 (2.80)
V (lower)	10 (2.00)
Family	
Nuclear	282 (56.40)
Joint	218 (43.60)
Marital status	
Married	341 (68.20)
Unmarried	147 (29.40)
Widow	12 (2.40)
Family	
Nuclear	282 (56.40)
Joint	218 (43.60)
Tobacco consumption	
Yes	224 (44.80)
No	276 (55.20)
Alcohol consumption	
Yes	137 (27.40)
No	363 (72.60)
Exercise	
Yes	151 (30.20)
No	349 (69.80)
Diet	
Vegetarian	297 (59.40)
Non-vegetarian	203 (40.60%)
Body mass index (kg/m^2^)	
<18.5 (underweight)	101 (20.20)
18.5–22.9 (normal weight)	194 (38.80)
23–24.9 (overweight)	194 (38.80)
>=25 (obese)	11 (2.20)
Height (m)^#^	1.65 ± 0.09
Weight (kg)^#^	60.63 ± 12.05
Body mass index^#^ (kg/m^2^)	22.10 ± 4.08

Table 2 depicts the domains of sleep from the PSQI. Almost half of the participants (242, 48.40%) had fairly good subjective sleep quality, while 114 (22.80%) had very good subjective sleep quality. More than half of the participants (290, 58.00%) had six to seven hours of sleep, and 133 (26.60%) had more than seven hours of sleep. Almost half of the participants (256, 51.20%) had a sleep efficiency of more than 85%. As for sleep disturbance, the maximum participants (370, 74.00%) experienced it fewer than once a week. Further, maximum participants (395, 79.00%) reported no use of sleep medication during the month preceding the data collection. Lastly, the median global PSQI score was 7 (5-9), and the maximum number of participants (214, 69.90%) were found to have poor quality of sleep (global PSQI score >5).

**Table 2:** Sleep quality among the study participants. ^*^: n=306. PSQI = Pittsburgh Sleep Quality Index

Sleep quality	N (%) (n = 500)
Subjective sleep quality	
Very good	114 (22.80)
Fairly good	242 (48.40)
Fairly bad	127 (25.40)
Very bad	17 (3.40)
Sleep duration (hours)	
>7 hours	133 (26.60)
6–7 hours	290 (58.00)
5–6 hours	46 (9.20)
<5 hours	31 (6.20)
Sleep efficiency	
>85%	256 (51.20)
75–84%	146 (29.20)
65–74%	51 (10.20)
<65%	47 (9.40)
Sleep disturbance	
Not during the past month	67 (13.40)
Less than once a week	370 (74.00)
Once or twice a week	61 (12.20)
Three or more times a week	2 (0.40)
Use of sleep medication	
Not during the past month	395 (79.00)
Less than once a week	69 (13.80)
Once or twice a week	24 (4.80)
Three or more times a week	12 (2.40)
Daytime dysfunction (score)	
0	198 (39.60)
1–2	214 (42.80)
3–4	79 (15.80)
5–6	9 (1.80)
Sleep latency* (score)	
0	9 (2.90)
1–2	125 (40.80)
3–4	147 (48.00)
5–6	25 (8.20)
Global PSQI score*	
<=5 (good quality sleep)	92 (30.10)
>5 (poor quality sleep)	214 (69.90)

The variables with p-values less than 0.25 on univariate logistic regression were entered into the multivariate logistic regression model to determine the predictors of poor sleep quality. The final model is depicted in Table 3 among 306 study participants, as the sleep latency domain was completed by 306 participants only. Only participants with complete data on PSQI were included in the multivariate logistic regression analysis. A PSQI score ≥5 indicates poor sleep quality. The mean age did not significantly differ between those with good (<5) and poor (≥5) sleep quality (40.60 vs. 40.81 years, p = 0.909). Gender-wise, a higher proportion of females (104, 74.8%) had poor sleep compared to males (110, 65.9%). Though this was not significant in the unadjusted analysis (p = 0.090), the adjusted OR (aOR) showed that females were significantly more likely to have poor sleep quality than males (aOR = 2.076, 95% CI = 1.141-3.776, p = 0.017). Urban and rural participants had similar sleep outcomes, with no significant difference in odds. Similarly, occupation and education level did not show any significant association with poor sleep quality in either unadjusted or adjusted models. There was no significant association between family structure (nuclear vs. joint) and sleep quality. Socioeconomic status also did not significantly predict poor sleep, though the data suggested some variability among classes. Unmarried or widowed individuals had higher rates of poor sleep (65, 75.6%) compared to married participants (149, 67.7%), but the association was not statistically significant in adjusted analysis (aOR = 1.605, p = 0.135). Tobacco use was a significant predictor. Tobacco users had higher odds of poor sleep (106, 75.2% vs. 108, 65.5%). While the unadjusted model was marginally non-significant (p = 0.065), the adjusted model revealed significantly higher odds among users (aOR = 2.470, 95% CI = 1.365-4.471, p = 0.003). Alcohol consumption, exercise, and diet type (vegetarian vs. non-vegetarian) were not significantly associated with sleep quality. Underweight participants (BMI <18.5 kg/m²) had significantly higher odds of poor sleep (38, 74.5%) compared to the obese category (51, 61.4%). In adjusted analysis, being underweight remained a significant predictor (aOR = 2.435, 95% CI = 1.029-5.762, p = 0.043). Higher depression scores (PHQ-9) were strongly associated with poor sleep. Those with moderate depression had an AOR of 2.643 (p = 0.033), and those with moderate-severe/severe depression had even higher unadjusted odds (OR = 9.577, p = 0.029), though the adjusted p-value slightly missed significance (p = 0.061). GAD-7 anxiety scores showed a significant unadjusted association for moderate anxiety (OR = 2.040, p = 0.049), but this was not significant after adjustment (aOR = 1.175, p = 0.702). A higher GERD-Q score (≥8) showed a trend toward poor sleep quality (73, 77.7% vs. 141, 66.5%) with marginal significance in the unadjusted analysis (p = 0.051) but was not significant after adjustment (p = 0.562).

**Table 3:** Factors predicting quality of sleep among the study participants. Model characteristics: Nagelkerke R-square = 0.149, Hosmer and Lemeshow test: p = 0.643. ^^^: per unit increase of age; ^*^: mean (standard deviation); ^$^: reference category; ^#^: row percentages. A p-value <0.05 is significant; unadjusted odds ratios (ORs) were calculated with univariate logistic regression; adjusted ORs were calculated using multivariate logistic regression. PSQI = Pittsburgh Sleep Quality Index; PHQ-9 = Patient Health Questionnaire-9; GAD-7 = Generalized Anxiety Disorder-7; GERD-Q = Gastroesophageal Reflux Disease Questionnaire; CI = confidence interval

Factors predicting quality of sleep	Global PSQI score (n = 306)		Unadjusted OR (CI)	P-value	Adjusted OR (CI)	P-value
	<5 n (%)^#^	>=5 n (%)^#^				
Age (^)* (years)	40.60 (14.05)	40.81 (15.19)	1.001 (0.985–1.018)	0.909	NA	NA
Gender						
Male	57 (34.10)	110 (65.90)	1^$^		1^$^	
Female	35 (25.20)	104 (74.80)	1.540 (0.935–2.536)	0.090	2.076 (1.141–3.776)	0.017
Residence						
Urban	56 (32.40)	117 (67.60)	0.775 (0.471–1.276)	0.317	NA	NA
Rural	36 (27.10)	97 (72.90)	1^$^			
Occupation						
Arithmetic, skilled, semi-skilled, semi-professional, unskilled	53 (32.10)	112 (67.90)	0.808 (0.494–1.323)	0.397	NA	NA
Retired, housewife, unemployed	39 (27.70)	102 (72.30)	1^$^			
Education						
Graduate, postgraduate	30 (30.30)	69 (69.70)	0.712 (0.334–1.515)	0.378	NA	NA
Higher secondary, high school, middle school, literate	49 (32.20)	103 (67.80)	0.651 (0.320–1.322)	0.235		
Illiterate	13 (23.60)	42 (76.40)	1^$^			
Family						
Nuclear	48 (29.10)	117 (70.90)	1^$^		NA	NA
Joint	44 (31.20)	97 (68.80)	0.904 (0.554–1.476)	0.688	NA	NA
Socioeconomic status						
I (upper), II (upper middle)	87 (29.90)	204 (70.10)	2.250 (0.251–20.131)	0.468	NA	NA
III (middle)	2 (25.00)	6 (75.00)	1.759 (0.385–8.023)	0.466	NA	NA
IV (lower middle), V (lower)	3 (42.90)	4 (57.10)	1^$^		NA	NA
Marital status						
Married	71 (32.30)	149 (67.70)	0.678 (0.384–1.196)	0.179	1^$^	
Unmarried/Widow	21 (24.40)	65 (75.60)	1^$^		1.605 (0.863–2.986)	0.135
Tobacco consumption						
Yes	35 (24.80)	106 (75.20)	1.598 (0.970–2.633)	0.065	2.470 (1.365–4.471)	0.003
No	57 (34.50)	108 (65.50)	1^$^		1^$^	
Alcohol consumption						
Yes	28 (28.90)	69 (71.10)	1.088 (0.641–1.845)	0.755	NA	NA
No	64 (30.60)	145 (69.40)	1^$^		NA	NA
Exercise						
Yes	22 (26.50)	61 (73.50)	1.269 (0.722–2.229)	0.408	NA	NA
No	70 (31.40)	153 (68.60)	1^$^		NA	NA
Diet						
Vegetarian	55 (30.70)	124 (69.30)	1^$^		NA	NA
Non-vegetarian	37 (29.10)	90 (70.90)	1.079 (0.656–1.774)	0.765	NA	NA
Body mass index (kg/m^2^)						
<18.5 (underweight)	13 (25.50)	38 (74.50)	2.573 (1.132–5.844)	0.024	2.435 (1.029–5.762)	0.043
18.5–22.9 (normal weight)	37 (30.60)	84 (69.40)	1.424 (0.792-2.563)	0.238	1.315 (0.702-2.465)	0.392
23–24.9 (overweight)	10 (19.60)	41 (80.40)	1.834 (0.850–3.959)	0.122	1.664 (0.710–3.899)	0.241
>=25 (obese)	32 (38.60)	51 (61.40)	1^$^		1^$^	
PHQ-9 score						
Minimal, mild	83 (34.70)	156 (65.30)	1^$^		1^$^	
Moderate	8 (16.70)	40 (83.30)	2.660 (1.190–5.947)	0.017	2.643 (1.083–6.448)	0.033
Moderate severe, severe	1 (5.30)	18 (94.70)	9.577 (1.256–73.007)	0.029	7.433 (0.911–60.643)	0.061
GAD-7 score						
Minimal, mild	80 (32.80)	164 (67.20)	1^$^		1^$^	
Moderate	11 (19.30)	46 (80.70)	2.040 (1.003–4.149)	0.049	1.175 (0.514–2.686)	0.702
Severe	1 (20.00)	4 (80.00)	1.951 (0.215–17.743)	0.553	1.147 (0.099–13.323)	0.913
GERD-Q score						
<=7	71 (33.50)	141 (66.50)	1^$^		1^$^	
>=8	21 (22.30)	73 (77.70)	1.750 (0.997–3.073)	0.051	1.208 (0.637–2.289)	0.562

## Discussion

Upper GI disorders and sleep disturbances are prevalent and often co-occurring health concerns. Prior research has identified a bidirectional relationship between GERD and sleep problems, including insomnia and poor sleep quality [25,26]. A bidirectional Mendelian randomization analysis has revealed a bidirectional relationship between GERD and insomnia. Further, upper GI disorders, including irritable bowel syndrome, are also correlated to anxiety and depressive disorders in varying degrees [27,28]. In light of this evidence, the present study attempted to study the prevalence of poor sleep quality and the relationship between sleep quality and upper GI symptoms in the Indian setting.

Almost two-thirds of the total participants (n = 306) in the present study reported poor sleep quality. Among those diagnosed with GERD, nearly 78% reported poor sleep quality. Although such a high prevalence was reported in our study, research elsewhere also reported that almost half or more than half of the patients with upper GI disorders experience sleep disturbances [29]. A cross-sectional study of 350 adult participants in Osaka reported that 53.9% of GERD patients were found to have sleep disturbances (on PSQI) compared to those who did not have upper GI symptoms. Further, this study also reported that anxiety and depression were more common in participants with sleep disturbances [30]. However, the multivariate logistic regression in this study did not identify GERD as a statistically significant predictor of poor sleep quality after adjusting for confounding factors (aOR = 1.208, p = 0.562). This finding emphasizes the need to consider other upper GI and psychosocial variables when evaluating sleep disturbances. In line with this statement, our study also identified sex, tobacco use, BMI, and depression as predictors of poor sleep.

There is evidence of a relationship between upper GI disorders and sleep disturbance at the molecular level. A case-control study conducted in China among GERD patients assessed its bidirectional relationship with sleep quality. Additionally, they reported that the transient receptor potential vanilloid type 1 receptor (TRPV1) in the esophageal mucosa could be a crucial player in disturbing sleep in GERD patients. Further, TRP channels are primarily responsible for assessing the physical and chemical milieu of the GI tract, which aids digestion. Any change in the functioning or expression of TRP channels results in GI disorders [31].

Given this, there has been an abundance of literature associating upper GI disorders with sleep disturbances. A community-based, cross-sectional study from Korea reported an association of sleep disturbances with digestive symptoms such as abdominal pain (aOR = 1.63) and reflux (aOR = 1.48) [32]. The present study also reported that GERD patients were 1.2 (aOR) times more likely to report poor sleep quality. A study from Taiwan assessing sleep disturbances and their association with gastric reflux reported that the former was independently predicted by older age, female gender, reflux symptoms, and depression [33]. The multivariable logistic regression model in the present study also reported female gender, tobacco consumption, low BMI, and moderate depression as significant predictors of poor sleep quality. Female gender was independently associated with higher odds of poor sleep (aOR = 2.076, p = 0.017), aligning with existing literature that reports greater sleep complaints among women [27,34]. Tobacco consumption also emerged as a significant contributor (aOR = 2.470, p = 0.003), potentially due to nicotine’s stimulating effects on the central nervous system and its role in aggravating GI symptoms such as dyspepsia and acid reflux [35,36]. Additionally, a population-based study evaluating the relationship between GERD and sleep reported that insomnia, sleeplessness, and problems falling asleep were three times more likely in participants with reflux symptoms. GER and poor sleep quality were also found to be associated with a study conducted among medical students in Iran [37]. The overall quality of life is also impaired by reflux symptoms [38-40]. Among other upper GI issues, a hospital-based study conducted in Beijing reported that consumption of tea, sweets, noodles, acidic food, overeating, lack of gap between dinner time and sleep, constipation, along with anxiety and depression, were found to be related to reflux esophagitis [35].

Depression and anxiety are also known independent predictors of poor sleep quality. Further, GERD also increases the risk of anxiety and depression along with sleep disturbances [41]. In the present study, approximately one-fifth of the participants had anxiety and depression. Participants with moderate depression (PHQ-9 scores of 10-14) had significantly higher odds of poor sleep quality (aOR = 2.643, p = 0.033). Although moderate anxiety (GAD-7 scores of 10-14) showed a significant association in unadjusted analysis, it was not significant in the adjusted model, possibly due to overlapping effects with depression. A study from Korea reported a higher frequency of sleep disruption in GERD cases than in the control group. Anxiety and depression were also found to be higher across variations of reflux diseases (erosive and non-erosive) [42]. The Men Androgen Inflammation Lifestyle Environment and Stress Study from Australia, conducted among 1,600 men, reported that participants with GERD were approximately three times more likely to have anxiety and twice as likely to have poor quality of sleep [43].

The above evidence is further reinforced by literature showing the efficacy of therapies for gastric reflux in reducing sleep disturbance. Drugs such as esomeprazole have resolved the sleep disturbances (on PSQI) related to GERD as well [44]. Fujiwara et al. conducted a study in Japan to estimate the efficacy of rabeprazole in GERD patients, where half of the patients reported a PSQI score of more than 5.5. Further, rabeprazole improved the symptoms and PSQI score after eight weeks [45]. On the other hand, insufficient sleep also increases the risk of developing nocturnal upper GI reflux, as reported by a longitudinal cohort study in adult women from Sweden [46].

There is a paucity of literature on psychological comorbidities related to upper GI disorders in India. However, the existing literature suggests that bed head elevation during sleep in nocturnal GER patients improved sleep disturbances in more than half of the patients [47]. Another study on risk factors of GERD in Indian medical students showed that inadequate sleep and lack of space between dinner and sleep were among other factors associated with GERD [48].

Study strengths

This is a first-of-its-kind study assessing sleep quality among patients reporting upper GI symptoms and the factors predicting poor sleep among these patients in the Indian settings.

Study limitations

A few variables, such as tobacco, alcohol consumption, and exercise, were self-reported and hence prone to underreporting due to social desirability bias. The wide CIs reported for the ORs of the moderate and severe depression categories are due to the small sample size in that category. The COVID-19 pandemic somewhat affected the rate of data collection. Moreover, there was a possibility of selection bias due to the study being conducted in a hospital setting. The pandemic might also have affected sleep patterns and hospital attendance. Lastly, unknown confounders could not be assessed in this study.

## Conclusions

Poor sleep quality is prevalent among patients with upper GI symptoms and is influenced by gender, lifestyle, nutritional status, and psychological well-being. Integrated care approaches using multicomponent interventions, including gastroenterologists, psychologists, and primary care specialists, targeted at specific risk groups, are essential for improving sleep health in this population. Counselling for mental health issues such as depression and tobacco use should be provided to patients visiting general outpatient clinics in hospitals, with complaints of upper GI symptoms. These should be addressed with a specific focus on the female gender.
